# Patients with stage IV colorectal carcinoma selected for palliative primary tumor resection and systemic therapy survive longer compared with systemic therapy alone: a retrospective comparative cohort study

**DOI:** 10.1097/JS9.0000000000001838

**Published:** 2024-06-27

**Authors:** Rene Mantke, Constanze Schneider, Anne von Ruesten, Michael Hauptmann

**Affiliations:** a Clinic for General and Visceral Surgery, University Hospital Brandenburg an der Havel, Brandenburg Medical School; b Faculty of Health Sciences Brandenburg, Brandenburg Medical School, Brandenburg; c Clinical-Epidemiological Cancer Registry Brandenburg Berlin, Cottbus; d Institute of Biostatistics and Registry Research, Brandenburg Medical School, Neuruppin, Germany

**Keywords:** colorectal neoplasms, palliative care, retrospective cohort study, survival analysis, systemic treatment

## Abstract

**Objective:**

To compare the survival of palliative stage IV colorectal cancer patients selected for primary tumor resection and systemic treatment (PTR+SYST) to patients with systemic treatment only (SYST).

**Background:**

About 20–25% of all colorectal cancer patients are diagnosed with stage IV disease. The benefit of primary tumor resection in the palliative situation is therefore of high concern. However, empirical evidence from randomized and observational studies is inconsistent.

**Methods:**

Mortality after PTR+SYST was compared to systemic treatment alone in a retrospective observational cohort of patients diagnosed 2012–2020 from the cancer registry in the federal state of Brandenburg (Germany), excluding patients with rectal cancer of the lower two-thirds, emergency procedures, unknown ECOG status, ECOG greater than 2, unknown metastatic status or unclear grading.

**Results:**

Of 480 patients, 416 died during an average follow-up of 23 months in mean. Twelve-month survival was 75% after PTR+SYST compared with 49% after SYST [hazard ratio (HR)=0.39, 95% CI 0.29–0.53, *P*<0.001]. The difference persisted to 36 months (28% vs. 13%, HR=0.53, 95% CI 0.43–0.66, *P*<0.001). Results were similar after multivariate adjustment, propensity score matching and delayed entry.

**Conclusion and relevance:**

Patients with stage IV colorectal carcinoma who are selected for primary tumor resection in combination with systemic therapy and who receive such treatment survive longer compared with patients who receive only systemic treatment. Whether the difference is due to the selection of patients or PTR remains unclear. At present, the current practice of selecting patients for PTR appears to do no harm.

## Introduction

Highlights

- Survival between Stage IV colorectal cancer patients with primary tumor resection with systemic treatment (PTR + SYST) and systemic treatment alone (SYST) was compared.
- PTR+SYST demonstrated significantly higher 12-month (75% vs. 49%, *P*<0.001) and 36-month survival rates (28% vs. 13%, *P*<0.001).

Colorectal cancer (CRC) is the third most commonly diagnosed cancer and the second most common cause of cancer-related death in the world^1^. In the United States more than 52 000 deaths in 2022 were estimated for colorectal cancer^2,3^. In Europe CRC is responsible for the second-highest number of cancer death in 2018^4^. However, the UICC tumor stage at diagnosis has a relevant influence on survival. About 20% of all colorectal cancer patients are diagnosed with stage IV disease (synchronous metastases)^3–5^. Only 16.2–20% of the stage IV patients with synchronous metastases undergo resection of the primary tumor and complete metastasectomy^5,6^. Therefore, screening efforts and treatment strategies for uncurable patients are of particular concern. Stage IV patients with a resectable primary tumor are selected in (a) resectable metastases, (b) initially unresectable metastases in whom resection may be possible after a major response following systemic therapy and in (c) unresectable metastases^4^. Different therapeutic approaches are recommended for each of the three groups. Primary tumor resection (PTR) for stage IV CRC patients was common even in patients with unresectable synchronous metastases but controversial after several prospective randomized controlled trials (RCT) observed no survival differences between patients treated with PTR followed by systemic treatment (PTR+SYST) compared with patients who received systemic treatment only (SYST)^7–10^. The current US and European ESMO guidelines do not recommend upfront PTR for unresectable metastatic CRC patients^4,11^.

On the other hand, several retrospective observational studies and RCTs comparing different chemotherapy regimens showed considerable survival advantages with PRT+SYST vs. SYST^6,12–17^. Discrepancies in the results between observational and randomized studies are not new in medicine in general and, in particular, in surgery^18–21^. It is known from several medical areas that randomized controlled trials can lack generalizability^22,23^. For this reason, it is important to evaluate registry data or other real-world data comprehensively.

However, the survival benefit observed for PTR+SYST over SYST alone in several observational studies was substantial^6,12–17^, even though most of these studies accounted for differences between the two groups in one way or another. If patients selected for PTR+SYST do actually benefit from PTR rather than just survive longer due to circumstances underlying their selection, the current practice of offering PTR to selected patients may do more good than harm.

Here, we present the results of a rather large cohort of stage IV CRC patients from the Brandenburg Berlin Cancer Registry. Registry data, although observational, can provide insights beyond those from RCTs, because if they cover an entire region, they include all patients and all treatment providers and offer what is often called a “real world” perspective^24–26^. On the other side, the lack of randomization often leads to indication bias which is hard to completely eliminate because what constitutes “indication” is often unknown or unobserved^27,28^.

Our objective was to use cancer registry data to compare the survival of patients with stage IV colorectal cancer with unresectable metastases who received upfront PTR+SYST with patients who received only SYST, using several measures to minimize indication bias.

## Data/methods

We evaluated data from all patients resident in the federal state of Brandenburg (Germany) and diagnosed with stage IV colorectal cancer according to UICC between 1 January 2012 and 31 December 2020 from the cancer registry for Brandenburg and Berlin. We included only patients with only systemic therapy (SYST) or with resection of the primary tumor and subsequent systemic therapy (PTR+SYST) and no documented tumor-free status (no complete resection of metastases). We excluded patients who had rectal cancer of the lower two-thirds, emergency procedures, unknown Eastern Cooperative Oncology Group (ECOG) performance status, ECOG greater than 2, unknown metastatic status or unclear grading (Fig. 2), that is the main analyses are based on complete cases.

PTR was defined as resection of the primary tumor within 12 months after diagnosis (OPS 5-455.*, 5-456.*, 5-484.*, 5-485.*). Cases with additional, but incomplete, resection of metastases were not excluded. SYST was defined as the application of a chemotherapy, antibody or immunotherapy (alone or in combination) starting within 12 months after diagnosis. The duration of systemic therapy was not taken into account. The endpoint was mortality. Only colon cancers without an indication for radiation were included in order to define the groups as homogeneously as possible. The present prospective randomized studies also mostly excluded rectal cancers that could also formally receive radiation.

Patient characteristics were compared between treatment groups using Pearson χ^2^ tests, Mann–Whitney U Test, and Fisher’s exact test. Survival of treatment groups (PTR+SYST vs. SYST) was compared using Kaplan–Meier survival plots and log-rank tests. Follow-up started at diagnosis and ended at death or 31 December 2020, whichever came first. Hazard ratios (HR) were calculated with multivariate Cox regression adjusting for sex (male, female), age at diagnosis (in years), ECOG status (0, 1, 2), localization and number of metastases according to TNM, 8^th^ edition (M1a, M1b, M1c), grading (G1–2, G3–4), localization of the primary tumor (colon carcinoma on the right side, colon carcinoma on the left side or rectum carcinoma upper third), and type and complications of systemic therapy.

Sensitivity analyses were performed to reduce indication bias. The effect of K-RAS mutations and of the location of metastases was evaluated outside the main analysis due to the large proportion of patients with unknown K-RAS status (~64%) and the sparse patient numbers in several of the 11 locations of metastases. In order to reduce indication bias, propensity score matching was conducted based on a propensity score determined by logistic regression of treatment (PTR+SYST vs. SYST) on the same variables as above plus radiotherapy (yes/no). For each patient in the SYST group, a patient from the PTR+SYST group was randomly chosen with the same propensity score than the SYST patient, within a certain margin. The same analyses as above were performed on the matched patient groups. Delayed entry was used to account for the fact that patients who underwent PTR must survive from the date of diagnosis to the date of surgery to be included in the PTR+SYST group, whereas no such requirement was made for patients in the SYST group. Follow-up started 3, 6, 9 and 12 months after diagnosis, excluding patients who died prior to this time irrespective of treatment (this method is called landmark by Alawadi *et al.* ^29^

*P* values less than 0.05 were considered statistically significant. Analyses were performed with SPSS (version 24, IBM) and R Studio (version 4.1.0, packages survival and survminer for Kaplan–Meier survival plots, package MatchIt for propensity score matching). The study has been reported in line with the STROCSS, Supplemental Digital Content 1, http://links.lww.com/JS9/C944 criteria^30^.

## Results

**Figure 1:**
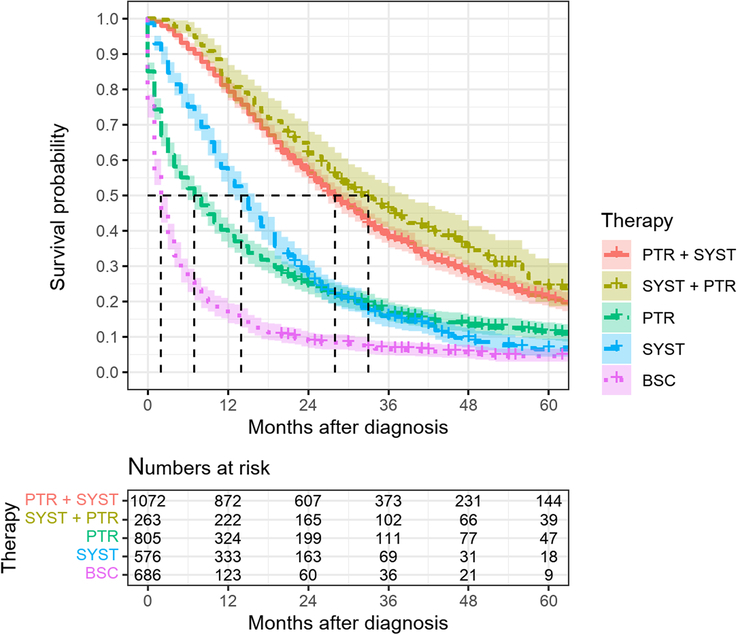
5-year absolute survival, colorectal cancer UICC stadium IV, *n*=3402, SYST+PTR group and PTR+SYST group include palliative patients and patients with complete tumor and metastatic resection (R0) within 1 year after diagnosis, 2012–2020. BSC, best supportive care, no resection of the primary tumor and no systemic therapy in the first year (*n*=686); PTR, resection of the primary tumor without systemic therapy in the first year (*n*=805); PTR+SYST, primary tumor resection and following systemic chemotherapy (*n*=1072); SYST, only systemic chemotherapy (*n*=576); SYST+PTR, primary tumor resection after systemic therapy (*n*=263).

**Figure 2:**
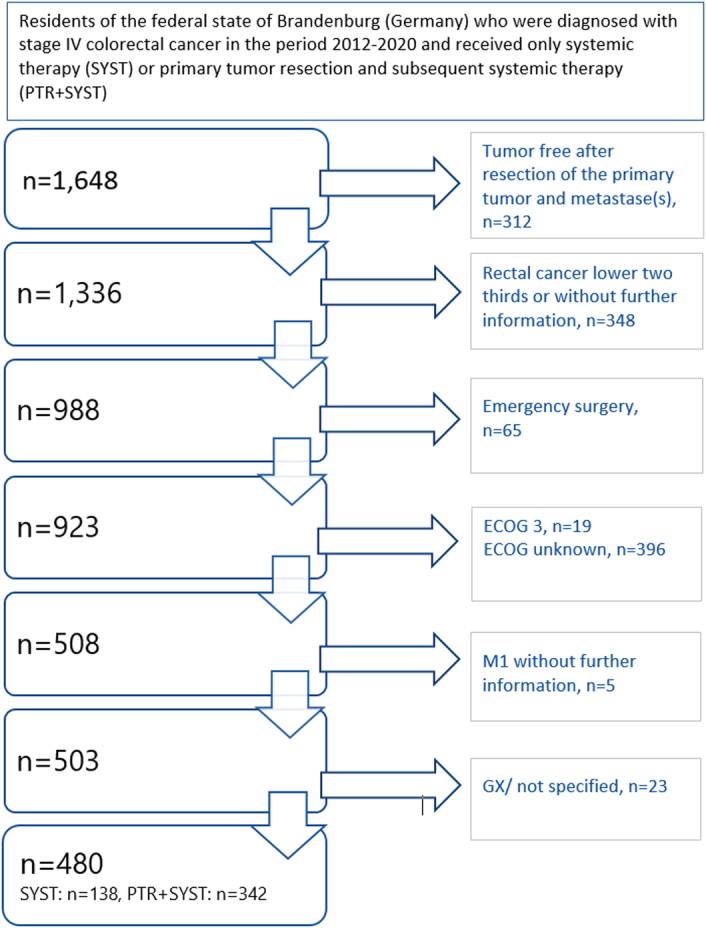
Selection of the study population. ECOG (Eastern Cooperative Oncology Group)– WHO Performance Status M1 without information on localization of the metastases/without specification M1a-c (according to TNM, 8th).

Between 2012 and 2020, 3402 patients were diagnosed with stage IV colorectal carcinoma and presented different survival data depending on the therapy regime (Fig. 1), including 1336 patients treated either with systemic therapy only or with palliative resection of the primary tumor and subsequent systemic therapy and no documented tumor-free status (Fig. 2). Of those, 480 patients met the inclusion criteria, 138 (28.75%) in the SYST group and 342 (71.25%) in the PTR+SYST group.

Patients in the PTR+SYST group were slightly younger, had a more advantageous metastases profile but higher grading compared to SYST (Figure S2, Supplemental Digital Content 2, http://links.lww.com/JS9/C945). In the SYST group 4 patients died within the first 30 days (2.9%) vs. none in the PTR+SYST group (Table 1). There were no significant differences concerning type, reason for termination or complications of the systemic therapy between the two groups (Table S3, Supplemental Digital Content 2, http://links.lww.com/JS9/C945, S4, Supplemental Digital Content 2, http://links.lww.com/JS9/C945). Synchronous metastases in the lungs and the lymph nodes occurred more often in the SYST group compared with PTR+SYST (Figure S2, Supplemental Digital Content 2, http://links.lww.com/JS9/C945).

**Table 1:** Patient characteristics according to treatment, *n*=480.

	Systemic therapy only, *n* (%)	Tumor resection (not tumor-free) and subsequent systemic therapy, *n* (%)	
	*n*=138	*n*=342	*P* ^a^
Sex			0.268
Male	91 (65.9)	207 (60.5)	
Female	47 (34.1)	135 (39.5)	
Age (years)			0.035
Median (IQR)	70.2 (61.5–77.7)	69.4 (59.3–75.7)	
ECOG			0.974
0	59 (42.8)	147 (43.0)	
1	54 (39.1)	136 (39.8)	
2	25 (18.1)	59 (17.3)	
Localization and number of metastases (according to TNM 8)	0.001		
M1a	54 (39.1)	186 (54.4)	
M1b	45 (32.6)	62 (18.1)	
M1c	39 (28.3)	94 (27.5)	
Grading			0.027
G1–2	108 (78.3)	233 (68.1)	
G3–4	30 (21.7)	109 (31.9)	
Localization of the primary tumor		0.654	
Colon carcinoma on the right side	51 (37.0)	119 (34.8)	
Colon carcinoma on the left side or rectum carcinoma upper third	87 (63.0)	223 (65.2)	
Death within 30 days			0.007
Yes	4 (2.9)	0	
No	134 (97.1)	342 (100.0)	

ECOG, Eastern Cooperative Oncology Group; IQR, interquartile range.

ECOG performance status: 0=normal, unrestricted (Carnovsky 90–100), 1=Limitation of physical exertion (Carn.70–80), 2=Self-sufficient but not able to work (Carn.50–60).

^a^ Pearson χ^2^ test (sex, ECOG, localization and number of metastases, grading, localization of the primary tumor), Mann–Whitney U Test (age), Fisher’s exact test (death within 30 days).

**Figure 3:**
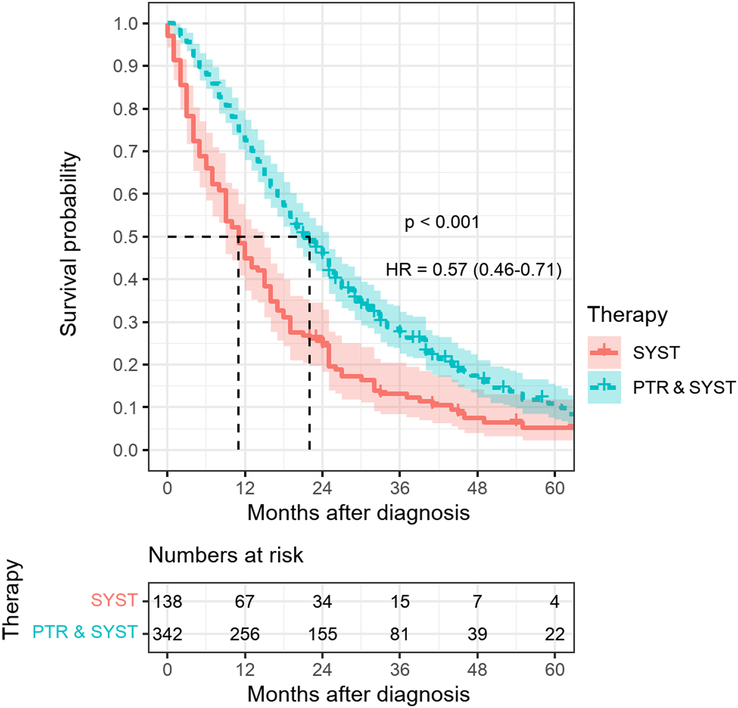
5-year absolute survival by type of treatment among stage IV colorectal cancer patients diagnosed 2012–2020, *n*=480, HR, hazard ratio; PTR & SYST, primary tumor resection followed by systemic therapy and no documented tumor-free status in the course (*n*=342); SYST, systemic chemotherapy only (*n*=138).

Among PTR+SYST patients with no documented tumor-free status in the course due to either unresectable or incompletely resected metastases, the primary tumor was completely resected in 80%, based on local R-status. The PTR+SYST group had significantly better absolute survival after 12 (75% vs. 49%), 24 (47% vs. 26%) and 36 (28% vs. 13%) months than the SYST group. Median survival was 11 months in the SYST group vs. 22 months in the PTR+SYST group (Fig. 3).

In the multivariate Cox regression, mortality was significantly reduced after PTR+SYST vs. SYST (HR=0.58, 95% CI: 0.46–0.71, Table 2). Other significant determinants of mortality were ECOG status (2 vs. 0), localization and number of metastases, and age.

**Table 2:** Association between treatment and all-cause mortality among patients with stage IV colorectal cancer based on multivariate cox regression.

				95% CI		
	*n*	No. deaths	Hazard ratio	Lower	Upper	*P*
Therapy (in the first year)						
Systemic therapy only	138	127	1			
Tumor resection (not tumor-free) and subsequent systemic therapy	342	289	0.58	0.46	0.71	<0.001
Sex						
Male	298	258	1			
Female	182	158	0.97	0.79	1.19	0.778
Age						
Per year			1.02	1.01	1.03	<0.001
ECOG						
0	206	168	1			
1	190	169	1.24	1.00	1.55	0.051
2	84	79	1.36	1.03	1.79	0.030
Localization and number of metastases (according to TNM 8)						
Metastases in one organ without peritoneal metastases (M1a)	240	201	1			
Metastases in two or more organs without peritoneal metastases (M1b)	107	100	1.48	1.15	1.90	0.002
Peritoneal metastases (M1c)	133	115	1.58	1.24	2.01	<0.001
Localization of the primary tumor						
Colon carcinoma on the right side	170	151	1			
Colon carcinoma on the left side or rectum carcinoma upper third	310	265	0.88	0.71	1.08	0.215
Grading						
1–2	341	288	1			
3–4	139	128	1.16	0.93	1.44	0.200

ECOG, Eastern Cooperative Oncology Group.

An impact on survival was shown for the group immunotherapy (better survival), but including the type and complications of systemic therapy does not change the estimated effect of the therapy in the first year (PTR+SYST vs. SYST).

In sensitivity analyses, proportions of patients with reported K-RAS mutations were similar between both groups (21.9% for PTR+SYST, 23.2% for SYST, about 64% missing data in both groups). Survival did not differ by K-RAS mutation status. Inclusion of K-RAS in the multivariate model (with a category for missing data) did not change the effect of PTR+SYST vs. SYST (not shown). Synchronous metastases at peritoneum, pleura, brain and lymph nodes were associated with a lower survival than other metastatic locations. However, when all metastasis locations are included, the effect of PTR+SYST vs. SYST is only slightly attenuated (HR=0.61, 0.49–0.77) and the association remains significant (not shown).

**Figure 4:**
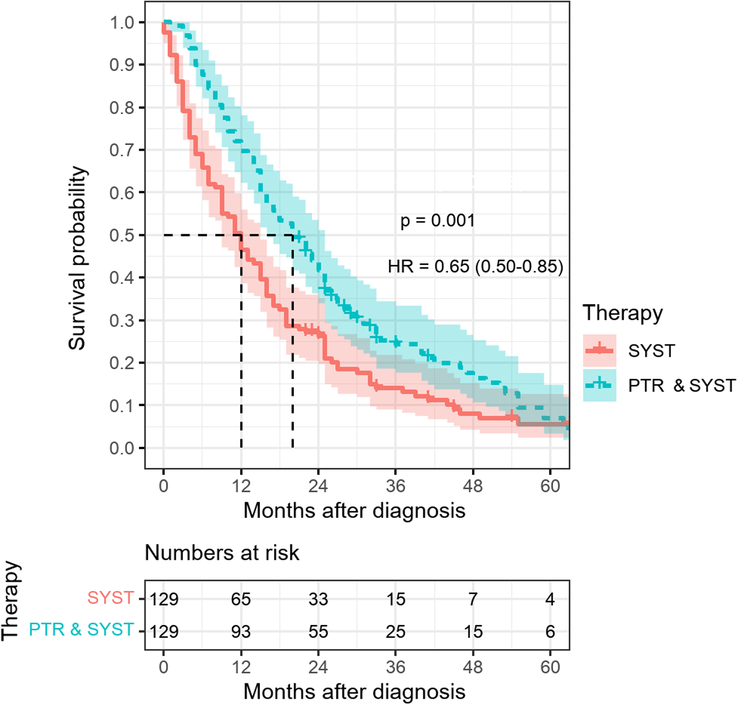
Absolute 5-year survival among stage IV colorectal cancer patients by treatment after propensity score matching. HR, hazard ratio; PTR & SYST, primary tumor resection and systemic treatment; SYST, only systemic chemotherapy.

With respect to confounding by indication multivariate adjustment just slightly attenuated the HR for PTR+SYST vs. SYST from 0.57 (95% CI 0.46–0.71) (Fig. 3) to 0.58 (95% CI: 0.46–0.71) (Table 2). Propensity score matching resulted in 129 patients after PTR+SYST matched with the same number of SYST patients (Table S1, Supplemental Digital Content 2, http://links.lww.com/JS9/C945, Figure S1, Supplemental Digital Content 2, http://links.lww.com/JS9/C945) and showed superior survival for PTR+SYST vs. SYST at 12 (72% vs. 50%), 24 (44% vs. 27%) and 36 months (25% vs. 14%) (Fig. 4). Median survival was 12 months for the SYST group and 20 months for PTR+SYST. The HR was 0.65 (95% CI 0.50–0.86, *P*=0.002) (Table S2, Supplemental Digital Content 2, http://links.lww.com/JS9/C945). Delaying start of follow-up by 3, 6, 9, and 12 months resulted in adjusted HRs of 0.64 (0.51–0.81), 0.71 (0.55–0.92), 0.72 (0.55–0.95), and 0.79 (0.59–1.08) (data not shown).

## Discussion

Our results show that palliative patients with stage IV colorectal carcinoma who are selected for primary tumor resection in combination with systemic therapy and who receive such treatment survive longer compared with patients who receive only systemic treatment.

The results are consistent with other retrospective analyzes and with a meta-analysis of four first-line chemotherapy trials^6,12–17^.

Faron *et al.* ^6^ described in a pooled analysis of 810 patients from four randomized first-line chemotherapy trials a better overall and better progression-free survival in the tumor resection group. Patients in the resection group had more colonic primaries, lower carcinoembryonic antigen (CEA) and alkaline phosphatase levels and more often a normal white-blood-cell count^6^.

Ahmed *et al.* ^12^ described 2016 a median overall survival from 18.3 month (PTR+SYST) vs. 8.4 month (only SYST) of course in a historic cohort treated from 1992-2005. The SYST group had more ECOG greater than 2 patients (38% vs. 22%), more rectal carcinomas, more extrahepatic disease, lower albumin levels and higher alkaline phosphatase levels. Symptomatic patients were more seen in the resection group (45% vs.28%)^12^.

Propensity scores are used in addition to a multivariate Cox proportional hazards analysis. Ishihara and colleagues found a significant positive effect of primary tumor resection on overall survival in a multivariate analysis where they also observed associations with calendar time, the institution of therapy and the extent of metastases. Survival remained superior after primary tumor resection in a propensity score-matched analysis, 13.8 vs. 6.3 months^17^.

Today, better systemic therapies and also targeted systemic therapies are available and are expected to improve survival in both groups. We were able to show that the advantage in survival for primary tumor resection with subsequent systemic therapy exists in more recent patient populations (12 vs. 20 months). In contrast, several RCTs found no difference in survival^7–10^. The results of two further prospective randomized studies (CLIMATE, SYNCHRONOUS) have not yet been fully published, although they have been closed for several years. Although RCTs produce the highest level of evidence for individual studies, they are very complex and often suffer from issues including high costs, slow recruitment, short follow-up, lack of generalizability, regulatory challenges, and lack of reproducibility^31^. Comparative effectiveness research evaluates the consequences of treatments in a broader context^31–33^. Therefore, the diverging results from observational studies supporting PTR cannot be easily dismissed.

Whether the selection of patients for PTR by the physician (and perhaps the patient) in the ward or PTR itself (or both) are responsible for superior survival cannot be answered by observational studies. Although the precise basis of the selection of patients for PTR is unclear, the data indicate that performing PTR in patients considered suitable for PTR plus systemic treatment. On the other side who would be assigned to the systemic treatment using which criteria?

To operationalize the decision for PTR in this patient group is not straight forward—it is probably a complex mixture of the physician’s experience, the patient’s condition and attitude, the local options and the guidelines. In a survey among international medical professionals, Tebala *et al.* ^34^ found that PTR is more often offered to younger patients with good performance status and in emergency situations. Currently available preliminary results of the CAIRO4 RCT suggest that patients who had elevated serum levels of lactate dehydrogenase, aspartate transaminase, alanine aminotransferase, and/or leukocytosis who were randomized to PTR had a significantly higher 60-day mortality than patients without these conditions^10^. Although these results could suggest that patients with transaminitis, leukocytosis, and primary tumors with obstructive symptoms should be considered for stenting rather than PTR, they are based on 60-day mortality and only 3 deaths^10^.

The fact that the currently used decision for PTR is unclear and cannot easily be operationalized means that we cannot offer rules for an indication for PTR in this patient group. It also means that the selection cannot be formally evaluated as a decision rule, because this would require an RCT which randomizes patients considered for PTR+SYST into PTR+SYST vs. SYST. Such a trial could shed light on whether the selection of patients can serve as a complex predictive biomarker for personalized medicine. Nevertheless, such a trial would require a formal definition of the decision for PTR, which is currently not available.

Our study has several strengths. The cancer registry has full coverage of the federal state of Brandenburg, including highly complete data on diagnosis, treatment and oncologic outcome. Since 2016, informed consent is not required for reporting cases. Patients have to opt out. Completeness case reporting is estimated to be greater than 90%^35,36^. In order to compare patients of the cancer registry with those of RCT, we attempted to use similar exclusion criteria. For example, many RCTs exclude lower and middle-third rectal cancers because radiotherapy is often administered. Or several RCTs exclude patients with symptoms of the primary tumor. Since we have no data on symptoms, we exclude patients who underwent emergency surgery. In particular, we tried to exclude similar patients as the prospective randomized SYNCHRONOUS study^8,9^. In addition, when we excluded patients with a prior history of cancer, we found similar results. As a sensitivity, we performed propensity score matching with similar results. When we delayed entry by 3, 6, 9, and 12 months, PTR-benefit was slightly attenuated but still significant. This indicates that patients in poor condition with short survival and therefore not selected for PTR are not driving our results.

Our analysis has some limitations. The registry data do not have detailed information on comorbidities, other factors characterizing patient fitness or MSI status. It was not possible to differentiate whether a relevant intestinal stenosis was present using the cancer registry data. By excluding cases with emergency operations, documented in the registry, a large proportion of patients with complete or almost complete stenosis or other acute problems such as bleeding or perforation should be excluded from our cohort.

We did not evaluate disease-specific survival because the cause of death is a complex and somewhat subjective mixture of underlying and contributing causes, including ICD-10 C18-C20 in the vast majority of deaths. Instead, we gave priority to the accurately and objective measured endpoint overall survival in our real-world data study.

In summary, because of the substantial survival benefit of PTR+SYST vs. SYST observed in several observational studies, even after propensity score matching and use of other bias reducing approaches, the current selection of a sizeable group of patients for tumor resection appears to help many patients to survive longer and, assuming the limited burden of surgery, seems a good choice. Since it may not be possible to ever unravel the precise basis of the decision for PTR, it is important to consider improvements in surgical methods to minimize morbidity by carrying out bowel resection with robotic assistance or minimally invasively, enrolling patients with PTR into an ERAS (Enhanced Recovery after Surgery) concept, conditioning malnourished patients preoperatively and performing operations without risky anastomoses while accepting a higher anus praeter rate. In addition, neo-adjuvant therapy is a promising approach to allow a better selection of patients for PTR after a response to systemic treatment.

## Ethical approval

Ethical approval was not required as the analyses are based only on anonymized patient data.

## Consent

Not applicable.

## Source of funding

This work was funded by the Ministry of Science, Research and Cultural Affairs of the State of Brandenburg, Germany.

## Author contribution

R.M.: conceptualization, formal analysis, investigation, methodology, project administration, resources, supervision, writing—original draft, writing—review and editing. C.S.: data curation, formal analysis, investigation, methodology, software, validation, visualization, writing—original draft. A.v.R.: data curation, formal analysis, investigation, methodology, resources, software, validation, visualization, writing—original draft, writing—review and editing. M.H.: conceptualization, formal analysis, investigation, methodology, project administration, resources, software, visualization, writing—original draft.

## Conflicts of interest disclosure

The authors declare no conflict of interests.

## Research registration unique identifying number (UIN)

Registry used: https://www.clinicaltrials.gov Unique Identifying number or registration ID: NCT06326619 Hyperlink to your specific registration: https://clinicaltrials.gov/study/NCT06326619

## Guarantor

Rene Mantke.

## Data availability statement

Data can be obtained from the Clinical Cancer Registry Berlin Brandenburg g GmbH upon reasonable request.

## Provenance and peer review

Not applicable.

## Supplementary Material

SUPPLEMENTARY MATERIAL

## References

[1] Sung H Ferlay J Siegel RL . Global Cancer Statistics 2020: GLOBOCAN estimates of incidence and mortality worldwide for 36 cancers in 185 countries. CA Cancer J Clin 2021;71:209–249. 33538338 10.3322/caac.21660

[2] Hernandez Dominguez O Yilmaz S Steele SR . Stage IV colorectal cancer management and treatment. J Clin Med 2023;12:2072. 36902858 10.3390/jcm12052072 PMC10004676

[3] Siegel RL Miller KD Fuchs HE . Cancer statistics, 2022. CA Cancer J Clin 2022;72:7–33. 35020204 10.3322/caac.21708

[4] Cervantes A Adam R Roselló S . Metastatic colorectal cancer: ESMO Clinical Practice Guideline for diagnosis, treatment and follow-up(†). Ann Oncol 2023;34:10–32. 36307056 10.1016/j.annonc.2022.10.003

[5] Väyrynen V Wirta EV Seppälä T . Incidence and management of patients with colorectal cancer and synchronous and metachronous colorectal metastases: a population-based study. BJS Open 2020;4:685–692. 32543788 10.1002/bjs5.50299 PMC7397359

[6] Faron M Pignon JP Malka D . Is primary tumour resection associated with survival improvement in patients with colorectal cancer and unresectable synchronous metastases? A pooled analysis of individual data from four randomised trials. Eur J Cancer 2015;51:166–176. 25465185 10.1016/j.ejca.2014.10.023

[7] Kanemitsu Y Shitara K Mizusawa J . Primary tumor resection plus chemotherapy versus chemotherapy alone for colorectal cancer patients with asymptomatic, synchronous unresectable metastases (JCOG1007; iPACS): a randomized clinical trial. J Clin Oncol 2021;39:1098–1107. 33560877 10.1200/JCO.20.02447 PMC8078424

[8] Rahbari NN Biondo S Feißt M . Randomized clinical trial on resection of the primary tumor versus no resection prior to systemic therapy in patients with colon cancer and synchronous unresectable metastases. J Clin Oncol 2022;40(17_suppl):LBA3507.

[9] Rahbari NN Lordick F Fink C . Resection of the primary tumour versus no resection prior to systemic therapy in patients with colon cancer and synchronous unresectable metastases (UICC stage IV): SYNCHRONOUS--a randomised controlled multicentre trial (ISRCTN30964555). BMC Cancer 2012;12:142. 22480173 10.1186/1471-2407-12-142 PMC3348093

[10] van der Kruijssen DEW Elias SG Vink GR . Sixty-day mortality of patients with metastatic colorectal cancer randomized to systemic treatment vs primary tumor resection followed by systemic treatment: the CAIRO4 phase 3 randomized clinical trial. JAMA Surg 2021;156:1093–1101. 34613339 10.1001/jamasurg.2021.4992 PMC8495602

[11] You YN Hardiman KM Bafford A . The American Society of Colon and Rectal Surgeons Clinical Practice Guidelines for the Management of Rectal Cancer. Dis Colon Rectum 2020;63:1191–1222. 33216491 10.1097/DCR.0000000000001762

[12] Ahmed S Leis A Fields A . Survival impact of surgical resection of primary tumor in patients with stage IV colorectal cancer: results from a large population-based cohort study. Cancer 2014;120:683–691. 24222180 10.1002/cncr.28464

[13] Allard MA Adam R . Patient with unresectable colorectal liver metastases and asymptomatic primary tumor: end of the debate!. Hepatobiliary Surg Nutr 2022;11:412–414. 35693410 10.21037/hbsn-22-176 PMC9186187

[14] Ferrand F Malka D Bourredjem A . Impact of primary tumour resection on survival of patients with colorectal cancer and synchronous metastases treated by chemotherapy: results from the multicenter, randomised trial Fédération Francophone de Cancérologie Digestive 9601. Eur J Cancer 2013;49:90–97. 22926014 10.1016/j.ejca.2012.07.006

[15] Galizia G Lieto E Orditura M . First-line chemotherapy vs bowel tumor resection plus chemotherapy for patients with unresectable synchronous colorectal hepatic metastases. Arch Surg 2008;143:352–358; discussion 358. 18427022 10.1001/archsurg.143.4.352

[16] Gresham G Renouf DJ Chan M . Association between palliative resection of the primary tumor and overall survival in a population-based cohort of metastatic colorectal cancer patients. Ann Surg Oncol 2014;21:3917–3923. 24859937 10.1245/s10434-014-3797-0

[17] Ishihara S Hayama T Yamada H . Prognostic impact of primary tumor resection and lymph node dissection in stage IV colorectal cancer with unresectable metastasis: a propensity score analysis in a multicenter retrospective study. Ann Surg Oncol 2014;21:2949–2955. 24763981 10.1245/s10434-014-3719-1

[18] Benson K Hartz AJ . A comparison of observational studies and randomized, controlled trials. N Engl J Med 2000;342:1878–1886. 10861324 10.1056/NEJM200006223422506

[19] Edwards JP Kelly EJ Lin Y . Meta-analytic comparison of randomized and nonrandomized studies of breast cancer surgery. Can J Surg 2012;55:155–162. 22449722 10.1503/cjs.023410 PMC3364302

[20] Lodi S Phillips A Lundgren J . Effect estimates in randomized trials and observational studies: comparing apples with apples. Am J Epidemiol 2019;188:1569–1577. 31063192 10.1093/aje/kwz100 PMC6670045

[21] Shikata S Nakayama T Noguchi Y . Comparison of effects in randomized controlled trials with observational studies in digestive surgery. Ann Surg 2006;244:668–676. 17060757 10.1097/01.sla.0000225356.04304.bc PMC1856609

[22] He Z Tang X Yang X . Clinical trial generalizability assessment in the big data era: a review. Clin Transl Sci 2020;13:675–684. 32058639 10.1111/cts.12764 PMC7359942

[23] Lim YMF Molnar M Vaartjes I . Generalizability of randomized controlled trials in heart failure with reduced ejection fraction. Eur Heart J Qual Care Clin Outcomes 2022;8:761–769. 34596659 10.1093/ehjqcco/qcab070 PMC9603541

[24] Banerjee R Prasad V . Are observational, real-world studies suitable to make cancer treatment recommendations? JAMA Netw Open 2020;3:e2012119. 32729916 10.1001/jamanetworkopen.2020.12119

[25] Blonde L Khunti K Harris SB . Interpretation and impact of real-world clinical data for the practicing clinician. Adv Ther 2018;35:1763–1774. 30357570 10.1007/s12325-018-0805-y PMC6223979

[26] Kumar A Guss ZD Courtney PT . Evaluation of the use of cancer registry data for comparative effectiveness research. JAMA Netw Open 2020;3:e2011985. 32729921 10.1001/jamanetworkopen.2020.11985 PMC9009816

[27] Bosco JL Silliman RA Thwin SS . A most stubborn bias: no adjustment method fully resolves confounding by indication in observational studies. J Clin Epidemiol 2010;63:64–74. 19457638 10.1016/j.jclinepi.2009.03.001 PMC2789188

[28] Streeter AJ Lin NX Crathorne L . Adjusting for unmeasured confounding in nonrandomized longitudinal studies: a methodological review. J Clin Epidemiol 2017;87:23–34. 28460857 10.1016/j.jclinepi.2017.04.022 PMC5589113

[29] Alawadi Z Phatak UR Hu CY . Comparative effectiveness of primary tumor resection in patients with stage IV colon cancer. Cancer 2017;123:1124–1133. 27479827 10.1002/cncr.30230 PMC5288308

[30] Mathew G Agha R Albrecht J . STROCSS 2021: Strengthening the reporting of cohort, cross-sectional and case-control studies in surgery. Int J Surg 2021;96:106165. 34774726 10.1016/j.ijsu.2021.106165

[31] Tunis SR Benner J McClellan M . Comparative effectiveness research: Policy context, methods development and research infrastructure. Stat Med 2010;29:1963–1976. 20564311 10.1002/sim.3818

[32] Conway PH Clancy C . Comparative-effectiveness research--implications of the Federal Coordinating Council’s report. N Engl J Med 2009;361:328–330. 19567829 10.1056/NEJMp0905631

[33] Hoffman A Montgomery R Aubry W . How best to engage patients, doctors, and other stakeholders in designing comparative effectiveness studies. Health Aff (Millwood) 2010;29:1834–1841. 20921483 10.1377/hlthaff.2010.0675

[34] Tebala GD Di Cintio A Ricci F . Primary tumour treatment in stage 4 colorectal cancer with unresectable liver and lung metastases and no peritoneal carcinomatosis-current trends and attitudes in the absence of clear guidelines. J Clin Med 2023;12:3499. 37240604 10.3390/jcm12103499 PMC10219093

[35] Cancer in Germany 2011/2012. Robert Koch Institut; 2015. 10.25646/3174

[36] Cancer in Germany 2013/2014. Robert Koch-Institut; 2017. 10.17886/rkipubl-2017-007

