# Supplementary material for: Patients with stage IV colorectal carcinoma selected for palliative primary tumor resection and systemic therapy survive longer compared with systemic therapy alone: a retrospective comparative cohort study
Source: Int J Surg. 2024 Jun 27;110(10):6493–500. doi: 10.1097/JS9.0000000000001838 (PMC11487045; doi:10.1097/JS9.0000000000001838)
Supplement: SUPPLEMENTARY MATERIAL [file js9-110-6493-s002.docx]

**Supplement**

Table S1: Baseline characteristics of patients with stage IV colorectal cancer, before and after propensity score (PS) matching

|  | **All patients (n=480)** | | | **PS-matched patients (n=258)** | | |
| --- | --- | --- | --- | --- | --- | --- |
| **Variable** | **Tumor resection (not tumor free) & subsequent systemic therapy (n=342)** | **Systemic therapy only (n=138)** | **Standard. Difference** | **Tumor resection (not tumor free) & subsequent systemic therapy (n=129)** | **Systemic therapy only (n=129)** | **Standard. Difference** |
| Propensity score (mean ± SD) | 0.73 ± 0.10 | 0.67 ± 0.12 | 0.61 | 0.69 ± 0.11 | 0.68 ± 0.11 | 0.11 |
| Males (%) | 60.5 | 65.9 | -0.11 | 65.9 | 65.1 | 0.02 |
| Age (years) (mean ± SD) | 67.1 ± 10.5 | 69.3 ± 10.8 | -0.21 | 68.2 ± 10.6 | 69.2 ± 10.6 | -0.09 |
| ECOG 0 (%) | 43.0 | 42.8 | <0.01 | 38.8 | 41.9 | -0.06 |
| ECOG 1 (%) | 39.8 | 39.1 | 0.01 | 42.6 | 41.9 | 0.02 |
| ECOG 2 (%) | 17.3 | 18.1 | -0.02 | 18.6 | 16.3 | 0.06 |
| M1a (%) | 54.4 | 39.1 | 0.31 | 44.2 | 41.1 | 0.06 |
| M1b (%) | 18.1 | 32.6 | -0.38 | 28.7 | 28.7 | 0.00 |
| M1c (%) | 27.5 | 28.3 | -0.02 | 27.1 | 30.2 | -0.07 |
| Colon carcinoma on the right side (%) | 34.8 | 37.0 | -0.05 | 35.7 | 36.4 | -0.02 |
| Colon carcinoma on the left side, Rectum carcinoma upper third (%) | 65.2 | 63.0 | 0.05 | 64.3 | 63.6 | 0.02 |
| Grading G1-2 (%) | 68.1 | 78.3 | -0.22 | 75.2 | 77.5 | -0.05 |
| Grading G3-4 (%) | 31.9 | 21.7 | 0.22 | 24.8 | 22.5 | 0.05 |
| Radiotherapy (%) | 3.8 | 5.8 | -0.10 | 4.7 | 4.7 | 0.00 |

ECOG performance status: 0=normal, unrestricted (Carnovsky 90-100), 1=Limitation of physical exertion (Carn.70-80), 2=Self-sufficient but not able to work (Carn.50-60)

Table S2: Association of therapy with all-cause mortality among propensity score-matched patients with stage IV colorectal cancer (n=258)

| **Therapy** | **n** | **Hazard Ratio** | **95% CI** | ***p*-value** |
| --- | --- | --- | --- | --- |
| Systemic therapy only | 129 | 1 |  |  |
| Tumor resection (not tumor free) & subsequent systemic therapy | 129 | 0.65 | 0.50-0.86 | 0.002 |

CI: Confidence Interval


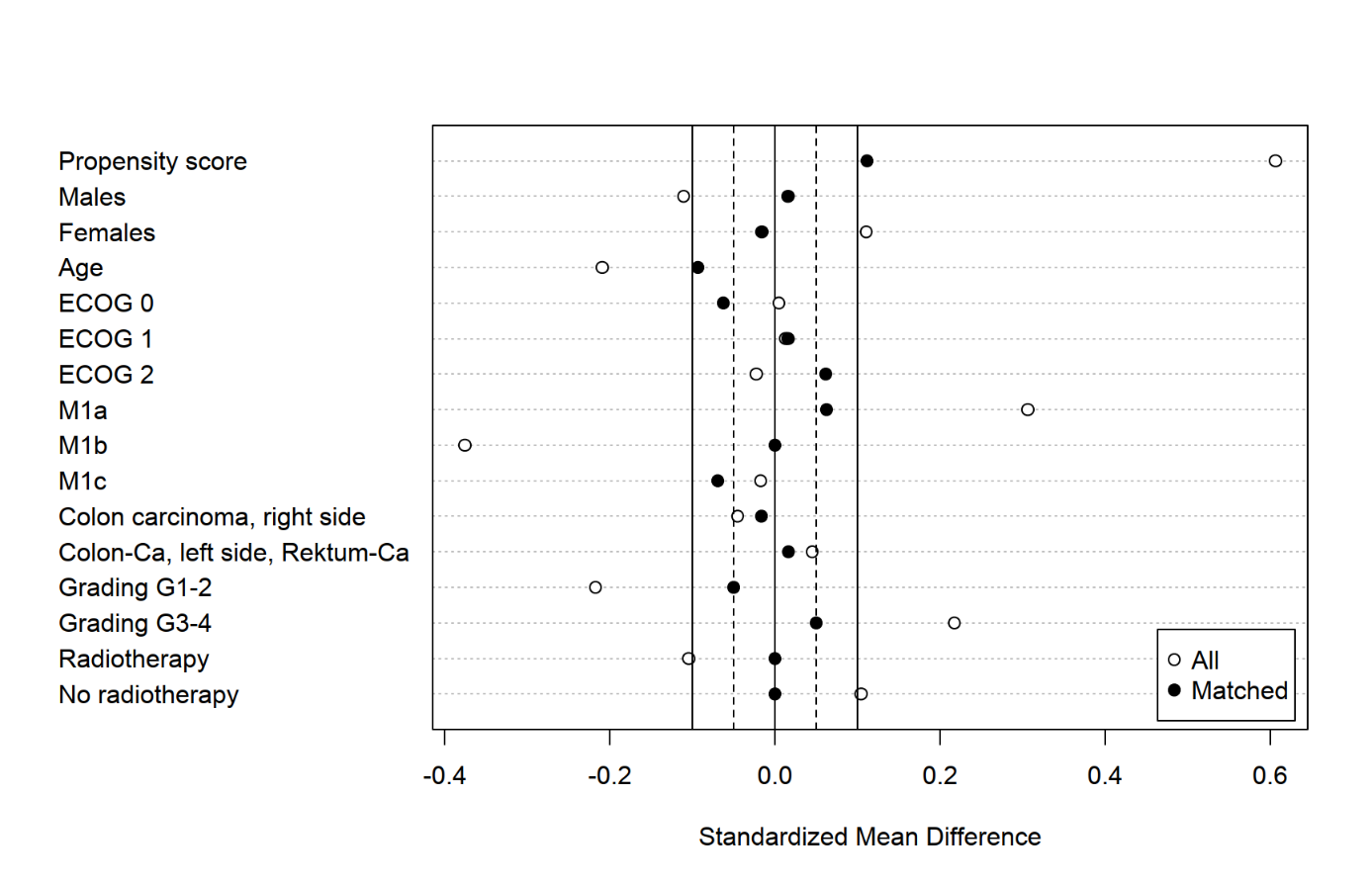


Figure S1: Balance of baseline characteristics of patients with stage IV colorectal cancer before and after propensity score matching

Figure S2: Localization of synchronous metastases (occurring within 92 days after diagnosis) according to treatment, n=480, more than one site possible

PTR & SYST: primary tumor resection followed by systemic therapy and no documented tumor free status in the course (n=342) SYST: systemic therapy alone (n=138)

* Significant difference between treatment groups

Table S3: Type of first systemic therapy among patients with stage IV colorectal cancer (n=480)

|  | **Systemic therapy only** | | **Tumor resection (not tumor free) & subsequent systemic therapy** | | **P Value*** |
| --- | --- | --- | --- | --- | --- |
|  | **n=138** | | **n=342** | |  |
| **Type of systemic therapy^1^** |  |  |  |  | **0.061** |
| **Chemotherapy** | **64** | **46.4%** | **127** | **37.1%** |  |
| Single agent | 6 | 4.4% | 14 | 4.1% |  |
| Combination of agents | 57 | 41.3% | 110 | 32.2% |  |
| Not specified | 1 | 0.7% | 3 | 0.9% |  |
| **Immunotherapy** | **74** | **53.6%** | **215** | **62.9%** |  |
| Immunotherapy alone | 5 | 3.6% | 2 | 0.6% |  |
| Chemo-Immunotherapy^2^ | 69 | 50.0% | 213 | 62.3% |  |

*Pearson chi-square test (comparison of chemotherapy vs. immunotherapy)

^1^First systemic therapy, subsequent change of protocol type is possible

^2^Including immunotherapy starting within 30 days after begin of chemotherapy

Table S4: Termination and complication rate of first systemic therapy among patients with stage IV colorectal cancer (n=480)

|  | **Systemic therapy only** | | **Tumor resection (not tumor free) & subsequent systemic therapy** | | **P Value*** |
| --- | --- | --- | --- | --- | --- |
|  | **n=138** | | **n=342** | |  |
| **Reason for termination of systemic therapy^1^** |  |  |  |  | 0.394 |
| Regular end | 25 | 18.1% | 83 | 24.3% |  |
| Termination due to side effects | 10 | 7.2% | 35 | 10.2% |  |
| Termination due to progress | 27 | 19.6% | 58 | 17.0% |  |
| Termination due to other reasons | 21 | 15.2% | 37 | 10.8% |  |
| Refusal of therapy by the patient | 4 | 2.9% | 6 | 1.8% |  |
| Unknown^2^ | 51 | 37.0% | 123 | 36.0% |  |
| **Documented complications of systemic therapy^1^** |  |  |  |  | 0.701 |
| Yes | 11 | 8.0% | 31 | 9.1% |  |
| no | 127 | 92.0% | 311 | 90.9% |  |

*Pearson chi-square test

^1^First systemic therapy, changes for subsequent therapies are possible

^2^Including cases with no documented end of systemic therapy
